# Anatomic evaluation of the height of the carotid bifurcation by 4^th^ year medical students using vascular ultrasonography

**DOI:** 10.1590/1677-5449.202401112

**Published:** 2025-04-18

**Authors:** Mateus Rodrigues Alessi, Murillo Campigotto Fedatto, Marcos Correa Segalla, Camila do Valle Pavanelo, Rodrigo Barberato, Gessil Dgeovani Carlotto, Graciliano José França

**Affiliations:** 1 Universidade Positivo – UP, Curitiba, PR, Brasil.; 2 Pontifícia Universidade Católica do Paraná – PUC-PR, Curitiba, PR, Brasil.; 3 Hospital Nossa Senhora das Graças, Curitiba, PR, Brasil.

**Keywords:** carotid arteries, anatomic variation, carotid artery ultrasound, students medical, teaching

## Abstract

**Background:**

The carotid bifurcation is known for its anatomical variations, involving structures that can be assessed by ultrasound examination. Knowledge of unusual anatomy is crucial in surgical procedures, directly influencing their outcomes.

**Objectives:**

To assess whether fourth-year medical students with prior training are capable of performing ultrasound examinations to compare the height of the carotid bifurcation between men and women.

**Methods:**

602 carotid bifurcations from 301 participants were identified by ultrasound examinations conducted by medical students after prior training by a professional qualified in vascular ultrasound. After each examination, the results were verified by a specialist physician. Gender, age, and bilateral measurement of the distance between the carotid bifurcation and the ear lobe were compared.

**Results:**

The students’ measurements differed from the specialist physician’s measurements by more than 0.2 cm in just 8 examinations. On the right side, the average height of the carotid bifurcation relative to the earlobe was 5.9 cm, compared to 5.8 cm on the left side, for the whole sample. The distance on the right side was significantly shorter among the women, with an average of 5.6 cm, compared to 6.3 cm among the men (p<0.0001). The distance on the left side was also significantly shorter in women, with an average of 5.4 cm, compared to 6.2 cm among the men (p<0.0001). The difference between sides was not statistically significant between the sexes.

**Conclusions:**

After training, medical students demonstrate high accuracy in the technique of measuring the carotid bifurcation height using vascular ultrasound. Men showed a tendency for the bifurcations to be located farther from the earlobe compared to women.

## INTRODUCTION

The majority anatomic variations of are found in the cardiovascular system. The term anatomic variations was officialized in 1543 in *De Humani Corporis Fabrica*. The majority are benign, but variations also encompass many pathological changes.^1^ For the majority of operating techniques, it is necessary to study and discover variations prior to surgical procedures, and doing so is directly related to achieving good outcomes. Some references consider that the term anatomic variations should not be used to avoid causing harm to people who have these variations, but this definition is controversial and used little in medical practice.^2,3^

Medical students are trained to memorize the usual anatomy, even though anatomic variations are widely found in the population, with the result that the most important morphological changes are often forgotten or even unknown to physicians.^4,5^ This educational error may be behind the causes of the majority of legal cases filed against surgeons, since 60% of surgical errors that lead to financial compensation for a patient or their family are due to surgical damage to peripheral nerves. Moreover, 27% of cases are related to inadvertent cutting or destruction of blood vessels, underscoring the importance of anatomic knowledge.^6,7^

There is consensus in the literature that the usual location of the carotid bifurcation is between vertebrae C3 and C4, or 5.9 cm from the earlobe in men and 5.3 cm from the earlobe in women. It has become convention that bifurcations above C3 are considered high and involve a greater degree of surgical risk in operations involving the head and neck.^8-12^ The most extreme data in the current literature describe bifurcations found at C1 and T4, which are variations with significant implications for surgical techniques.^13^ It is also important to mention that, while it is commonplace to study the height of the bifurcation of the carotid artery (BCA) measured from the earlobe, there are studies that use the hyoid bone or the angle of the mandible in an attempt to increase measurement accuracy, since the earlobe is soft tissue and grows as humans age.^14^ Nevertheless, the earlobe was chosen as anatomic marker for the present study, in reference to the majority of articles available, and because this is the most widely accepted site in the literature.^9,10^

While there are many studies assessing medical students’ competence to conduct ultrasound (US) examinations, there are no articles assessing their performance when assessing the carotid artery.^15-19^ As such, the objective of this study is to evaluate whether fourth-year medical students with prior training are able to correctly measure the height of the carotid bifurcation using a portable US machine.^15,20,21^ Additionally, the article also presents statistical analyses of the measurements obtained comparing males and females.

## METHODS

This is a non-interventional, observational study attempting to compare variations in the height of the carotid bifurcation bilaterally in male and female subjects. The study also investigated whether medical students trained for 60 hours would be capable of identifying and correctly measuring the height of the carotid bifurcation, obtaining similar results to professional vascular ultrasound operators.

**Figure 1:**
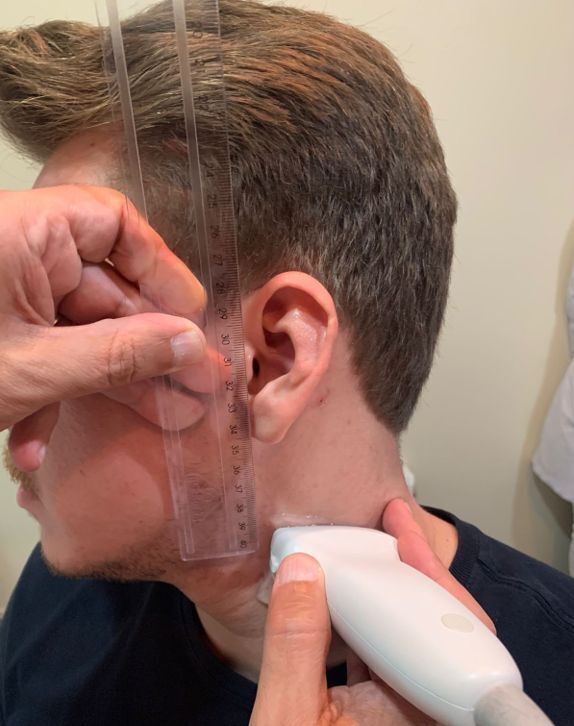
Image illustrating how the position of the carotid bifurcation was measured in relation to the earlobe.

Over a 1-month period, six fourth-year medical students studying at a university in the state of Parana, Brazil, took part in classes on portable vascular ultrasonography. Lessons were given by two vascular surgeons from the same state, with a total of 40 hours of theory and 20 hours of practical training. After training, an individual test was conducted with 15 volunteers, in which each student examiner performed the technique for measuring the distance from the earlobe to the site at which the common carotid artery gives rise to the external and internal carotid arteries and, at the end, their measurements were compared to those obtained by the teacher (Figure 1). It was pre-established that two or more discrepancies exceeding 0.3 cm or one discrepancy greater than or equal to 0.5 cm would invalidate that researcher from participating in data collection. At the end of the validation test, all six participants were considered fit to participate. The ideal number of examiners indicated that just two participants would be sufficient, but it was decided to include all six students, for greater learning.

**Figure 2:**
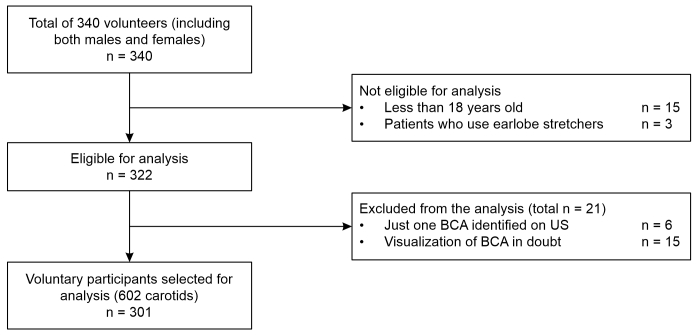
Flowchart showing inclusion, exclusion, and losses of study participants. BCA = bifurcation of the carotid artery; US = ultrasound.

After the initial training phase, a sample size calculation indicated that at least 280 volunteers were needed for the study. The group of students therefore performed the same measurement technique on 301 participants: all seated, in an erect position, with the head slightly tilted. The patient’s position was defined after a pretest which did not detect any difference between measurements made with the patient sitting or lying down. Participants were required to remove any earrings located in the earlobe and patients with stretched earlobes were excluded from the sample (Figure 2). It was decided that throughout the study all measurements made by the students would be compared to the results of measurements made by a professional vascular surgeon qualified in vascular echography, who would be waiting in a separate room and would be blind to the students’ results. If any discrepancies exceeding 0.2 cm were observed, another medical professional, also a specialist in vascular ultrasonography, would be requested to measure the participant a third time. If the two physicians’ measurements differed by more than 0.2 cm, these two results would be summed and divided by two to give a mean distance.

It is important to point out that both of these physicians were vascular surgeons and both were certified to perform vascular echography with Doppler by the Brazilian College of Radiology and the Brazilian Society of Angiology and Vascular Surgery.

All of the 301 participants were selected at random using statistical software that attributed a number to each of the students at a university in Parana, Brazil, at random, respecting the criterion of voluntary participation, with agreement to a free and informed consent form and prior approval by the Ethics Committee (Ethics Appraisal Submission Certificate: 60822422.6.0000.0093 and Approval Number: 5.997.677). Data entry was sequential, by date and order of examination. Additionally, both carotid bifurcations of each participant were measured and included in the study.

It was pre-established that in cases in which only one BCA was identified or in which visualization was in doubt, the data would be excluded from the analysis.

**Figure 3:**
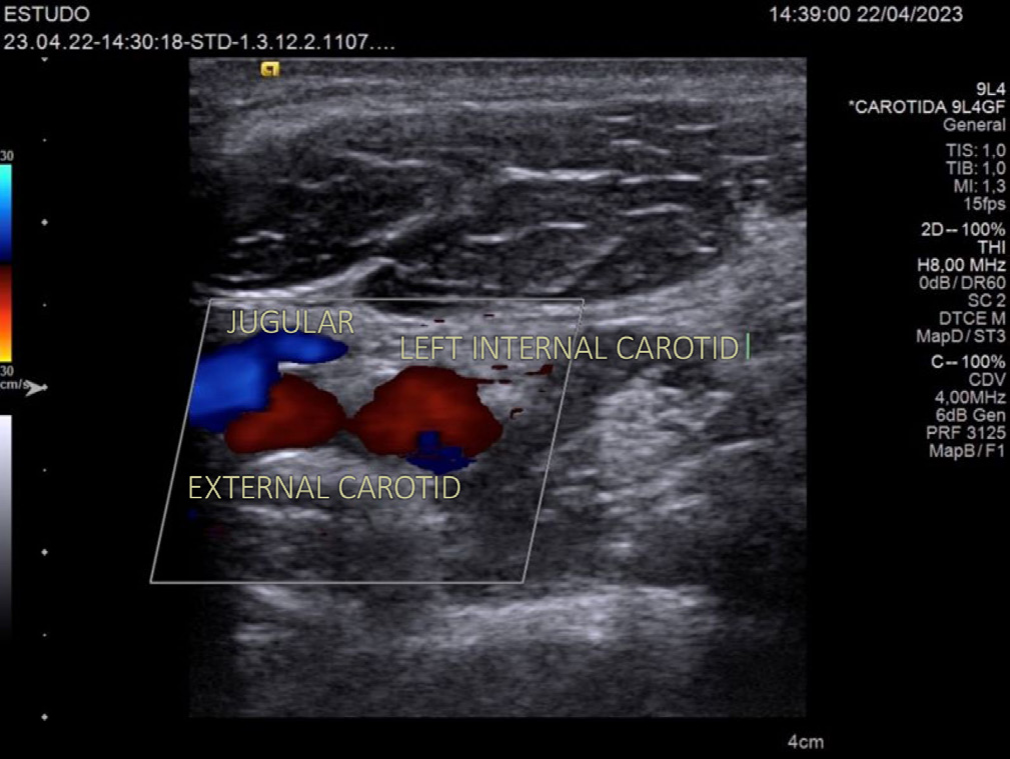
Transverse color Doppler flow ultrasound image showing the left internal carotid and left external carotid arteries and the left jugular vein.

**Figure 4:**
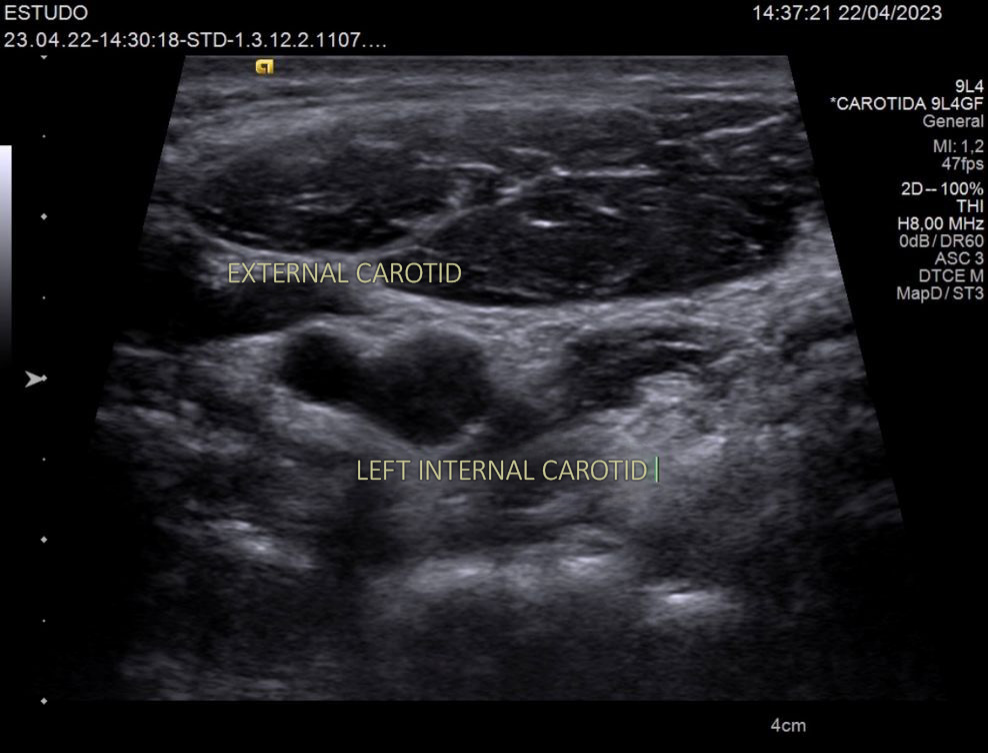
Transverse ultrasound image showing the left carotid bifurcation. The left external carotid artery and left internal carotid artery are being analyzed.

All carotid arteries were examined using the same portable US machine, Phillips brand, Lumify model, using a linear probe. The technique involved identifying the common carotid artery and following its path distally until the origin of the external and internal carotid arteries was located. The point immediately proximal of the origin of these two vessels was used as the measurement point. Compression maneuvers would be performed if the identity of veins and arteries was unclear (Figures 3 and 4).

Participants’ initials and sex were recorded in addition to their aged and the bilateral bifurcation height measurements. The eight cases in which the students’ and echography specialists’ measurements differed by more than 0.2 cm were also noted. Student’s *t* test was used to compare quantitative variables and Fisher’s test was used for categorical variables. Statistical significance was defined as p < 0.05.

## RESULTS

During the course of the study, just eight of the 602 measurements needed the second medical professional to take a second measurement. When done, in all cases the two professional physicians’ measurements differed by 0.2 cm or less, so there was no need to take the mean of their results.

Of the total sample of 602 carotid bifurcations measured (301 individuals), 332 were in women (55.1%) and 270 in men (44.9%). The mean age of the participants was 23.2 years, with 245 individuals (81.4%) aged 18 to 25 years and 56 (18.6%) older than 25 years. The mean height of the carotid bifurcation on the right side was 5.9 cm, whereas the height on the left side was 5.8 cm, with a mean difference between sides of 0.8 cm (Table 1).

**Table 1:** General characteristics of the study.

**General characteristics (n = 301)**		
Sex	**n**	**%**
Female	166	55.1
Male	135	44.9
Age brackets (years)		
18-25	245	81.4
>25	56	18.6
Age (years)		
Mean ± SD	23.2	± 3.7
Right		
Mean ± SD	5.9	± 1.3
Left		
Mean ± SD	5.8	± 1.2
Difference		
Mean ± SD	0.8	± 0.1

SD = standard deviation.

The distance from the earlobe to the carotid bifurcation was longer in men than in women. On the right side, the mean distance was 5.6 cm in women and 6.3 cm in men (p < 0.0001). On the left side, the mean distance was 5.4 cm in women and 6.2 cm in men (p < 0.0001). The largest distance observed was in a male participant, at 9.5 cm, and the smallest was 3 cm, in a female participant. The variation in distance between the two sides was not statistically significantly different between the sexes (p = 0.776) (Table 2). The largest difference observed was in a woman, with 8.5 cm on the left side and 4 cm no right side. Analysis of participants’ ages, divided into two groups (18-25 years and > 25 years), did not detect any significant results for the variation in the height of the bifurcation (p = 0.052).

**Table 2:** Comparison between females and males.

	**Females**		**Males**		**p**
	**Mean**	**± SD**	**Mean**	**± SD**	**0.037**
Age (years)	22.4	3.6	24.3	3.8	< 0.0001
Right	5.6	1.1	6.3	1.3	< 0.0001
Left	5.4	1.1	6.2	1.3	0.776
Difference	0.7	0.1	0.8	0.1	
Age brackets (years)	n	%	n	%	
18-25	142	85.5	103	76.3	0.052
> 25	24	14.5	32	23.7	

SD = standard deviation.

## DISCUSSION

Although none of the six medical students who took part in the study have vascular certification, they were capable of obtaining BCA measurements that were very similar to those obtained by trained and qualified professionals. This result was due to intense training, both theoretical and practical, totaling 60 hours, in addition to their individual dedication. This result feeds the discussion that ultrasonography is not an examination technique that should only be taught to trained physicians or during medical residency that includes it in the curriculum. However, Brazil’s Ministry of Education and Culture does not mandate a minimum number of teaching hours on echography in medical schools, with the result that many physicians qualify without becoming familiar with performing simple ultrasonographic examinations that very often take a few minutes, but can change the patient’s clinical outcomes, especially in intensive care (ICU) or emergency settings.^22-25^ In contrast, the many universities around the world that do include training in ultrasonography in their curricula observe significant improvements in learning, motivation, and physical examination accuracy when training with US is included in both the clinical and the anatomic components of medical degrees.^16-18,26-28^

The finding that previously trained medical students are able to measure the height of the BCA with comparable precision to experienced professionals has limited practical applicability. This is because this measurement is just one part of the carotid artery ultrasonography protocol and these students are not certified to write reports for this examination. However, the study does raise questions about what other examinations these students could perform with satisfactory precision. While the technique for measurement of the height of the BCA is simple, it demands anatomic knowledge, which is also crucial for identification of the internal jugular veins. This is an essential step in fitting US-guided central venous access, which is an advanced procedure that can be performed by physicians with no specific certification.

Brazil is a highly heterogeneous country, with shortages of material resources, qualified professionals, and imaging exams, especially away from the large urban centers. Physicians who practice in regions in the country’s interior are often faced with a lack of adequate support. While the ideal would be to have a specialist ultrasound operator available to conduct examinations, this is not always feasible in remote areas. It is therefore proposed that newly-qualified physicians should be trained to perform basic ultrasound examinations in emergency situations, primarily in places where there are no other qualified professionals available. These examinations can be decisive for patient’s clinical outcomes and in many cases they constitute the only diagnostic option available because of lack of resources.^22-25^

One cannot discuss US without commenting on bedside US techniques, or point-of-care ultrasound (POCUS), which consists of conducting the ultrasonographic examination in the patient’s current location, whether in the ICU or even at their home, for rapid diagnoses and assessments. Its speed and ability to produce findings that change patient management has led to its being incorporated more and more into medical practice. The examinations performed for this study are compatible with this technique, meaning that this article is another data point demonstrating that medical students are highly capable of performing them and interpreting the images.^19,29-31^ It is probable that bedside US will become a requirement of medical training in the not-too-distant future. Many different specialty certification tests, in Brazil and beyond, already include a POCUS examination as part of the appraisal. Moreover, many physicians already believe that as ultrasound machines, which can already be connected to smartphones or tablets, develop use of this procedure will become another pillar of the physical examination, in conjunction with inspection, palpation, percussion, and auscultation.^32^

Analysis of the results for measurements of the height of the BCA revealed that they were similar to those reported in the majority of studies available in the international and Latin American literature, including those that employed ultrasonography, computed tomography angiography, cadaveric dissection, and surgical incision.^8-10,13,33,34^ Observing only the results of those studies that included other anatomic landmarks as reference to assess the height of the BCA, the results were also similar when it was determined to which cervical vertebra the distances reported referred. This is therefore one more study corroborating the idea that the majority of BCAs are found between C3 and C4 and that females tend to have higher bifurcations, i.e., more distal in relation to the origin of the common carotid. It is valid to point out that the participants’ ages did not demonstrate any significant influence on the distance to the BCA, in common with all of the other studies analyzed. The similarity of the results, especially to studies undertaken in different populations, such as from Latin America, is important considering that human morphology can vary between populations because of ethnic and geographic differences.^14,33,35^ These results, similar to those in the literature, underscore the idea that the measurement technique used by the medical students was correct.

The true importance of knowing where the BCA is located is primarily observed in surgical procedures, in particular endarterectomy, catheterizations, and radical neck dissections.^14,35-37^ Its greatest relevance is for predicting the difficulty, or even impossibility, of performing endarterectomy, a surgery that consists of removing atheromatous plaques from the carotid walls and which is considered the gold standard treatment for carotid stenosis. This technique is not recommended for bifurcations higher than C2, for which stenting is preferred.^14,34-38^ Therefore, one of the most important reasons for determining the height of the BCA and knowing which anatomic variations may occur at this site is that higher bifurcations might demand alternative techniques or even displacement of the mandible during surgery.^39^ It is worth noting that while female participants had a shorter distance from the earlobe to the BCA, no bifurcations above the C3 were observed.

Certain questions that merit reflection involve the practical and economic justifications for training medical students to conduct US examination that require specialist training and the precision of which is highly dependent on examiner experience, with final results that are not therefore guaranteed to be reliable. Questions may also be asked about the relevance of teaching these students to identify the height of the carotid bifurcation, which is knowledge that is of more specific interest to vascular surgeons. Additionally, little is known about the true value of including the study of rare anatomic variations in the medical curriculum, given their limited applicability in clinical practice.

This study has several limitations. The first is the mean age of the participants (23.2 years), with no participants at the extremes of age. Another point that merits mention is that the study did not collect data such as: body mass index, height, or body surface area, which could be considered confounding factors or effect modifiers. Moreover, the fact that the earlobe depends on several factors to yield a precise measurement, (such as the posture of both examiner and subject examined and anatomic or genetic differences), there may be an increased likelihood of data collection errors, particularly since measurements were taken by undergraduate medical students.

## CONCLUSIONS

In conclusion, after appropriate training, medical students are capable of performing precise measurements of the height of the BCA using portable ultrasonography. With regard to the measurements obtained, women tend to have carotid bifurcations closer to the earlobe than men, and the difference between the two sides was not significantly different between the sexes.
