# Supplementary material for: Anatomic evaluation of the height of the carotid bifurcation by 4th year medical students using vascular ultrasonography
Source: J Vasc Bras. 2025 Apr 18;24:e20240111. doi: 10.1590/1677-5449.202401112 (PMC12020742; doi:10.1590/1677-5449.202401112)
Supplement: Translation

# Translation: Avaliação anatômica da altura da bifurcação carotídea com ultrassonografia vascular portátil por alunos de medicina do quarto ano

**DOI:** 10.1590/1677-5449.202401111

**Authors:** Mateus Rodrigues Alessi, Murillo Campigotto Fedatto, Marcos Correa Segalla, Camila do Valle Pavanelo, Rodrigo Barberato, Gessil Dgeovani Carlotto, Graciliano José França

**Affiliations:** 1 Universidade Positivo – UP, Curitiba, PR, Brasil.; 2 Pontifícia Universidade Católica do Paraná – PUC-PR, Curitiba, PR, Brasil.; 3 Hospital Nossa Senhora das Graças, Curitiba, PR, Brasil.

**Keywords:** artérias carótidas, variação anatômica, ultrassonografia das artérias carótidas, estudantes de medicina, ensino

## Abstract

**Contexto:**

A bifurcação carotídea é um local conhecido por variações anatômicas, cujas estruturas podem ser avaliadas por exames de ultrassom. O conhecimento da anatomia não usual é crucial em procedimentos cirúrgicos, influenciando diretamente seus resultados.

**Objetivos:**

Avaliar se estudantes de Medicina do quarto ano, previamente treinados, são capazes de realizar exames ultrassonográficos comparando a altura da bifurcação carotídea entre homens e mulheres.

**Métodos:**

No total, 602 bifurcações carotídeas de 301 participantes foram identificadas por ultrassom pelos alunos, após treinamento prévio por um profissional habilitado em ecografia vascular. Após cada exame, os resultados foram verificados por um médico especialista. Comparou-se sexo, idade e medida bilateral da distância entre a bifurcação carotídea e o lóbulo da orelha.

**Resultados:**

Apenas oito exames diferiram em mais de 0,2 cm entre a medida dos alunos e do médico especialista. À direita, a altura média da bifurcação carotídea em relação ao lóbulo da orelha foi de 5,9 cm, e à esquerda, 5,8 cm. A distância à direita foi significativamente menor em mulheres, com média de 5,6 cm, enquanto nos homens, a média foi de 6,3 cm (p < 0,0001). A distância à esquerda também foi menor em mulheres, com média de 5,4 cm, enquanto em homens foi de 6,2 cm (p < 0,0001). As diferenças entre os lados não foram estatisticamente significativas entre os sexos.

**Conclusões:**

Após treinamento, estudantes de Medicina demonstraram precisão elevada na técnica de medida da altura da bifurcação carotídea por ecografia vascular. Homens apresentaram tendência a bifurcações mais distantes do lobo da orelha em comparação às mulheres.

## INTRODUÇÃO

O sistema cardiovascular é o que apresenta a maior parte das variações anatômicas, tendo este termo sido oficializado em 1543 na obra *De Humani Corporis Fabrica*. A maioria dessas alterações são benignas, mas abrangem grande parte de alterações patológicas^1^ . Seu estudo e conhecimento prévio a procedimentos cirúrgicos é necessário na maioria das técnicas operatórias, estando diretamente relacionado a um bom desfecho. Algumas referências compreendem que o termo variações anatômicas não deve ser utilizado na eventualidade de causar malefícios aos seus portadores, porém essa definição é controversa e pouco utilizada na prática médica^2,3^ .

Estudantes de Medicina são treinados para memorizarem a anatomia usual, mesmo com as variações anatômicas sendo amplamente presentes na população, o que faz com que as principais alterações morfológicas sejam frequentemente esquecidas ou desconhecidas pelos médicos^4,5^ . Esse erro educacional pode estar por trás do motivo da maioria dos processos legais contra médicos cirurgiões, uma vez que 60% dos erros cirúrgicos que culminaram em compensação monetária ao paciente ou sua família decorreram de danos cirúrgicos em nervos periféricos. Além disso, 27% dos casos estiveram relacionados a cortes ou destruições indevidas de vasos sanguíneos, ressaltando a importância do conhecimento anatômico^6,7^ .

Há consenso na literatura de que o local habitual da bifurcação carotídea se encontra entre as vértebras C3 e C4, ou à 5,9 cm do lobo da orelha em homens e 5,3 cm em mulheres. Foi convencionado que bifurcações superiores à C3 são consideradas altas, apresentando maiores riscos cirúrgicos em operações envolvendo a cabeça e pescoço^8–12^ . Os dados mais extremos presentes na literatura atual apontam que bifurcações já foram encontradas em C1 e T4, variantes que teriam implicância significativa durante técnicas cirúrgicas^13^ . É importante ressaltar que, embora seja comum estudar a altura da bifurcação da artéria carótida (BAC) a partir da posição do lobo da orelha, existem estudos que utilizam o osso hioide ou o ângulo da mandíbula em uma tentativa de aumentar a acurácia das medidas, visto que a orelha é um tecido mole e que cresce de acordo com o envelhecimento humano^14^ . Apesar disso, o presente estudo decidiu utilizar o lobo da orelha como marcador anatômico, tendo como referência a maioria dos artigos disponíveis, além de ser esse o local mais consagrado na literatura^9,10^ .

Mesmo já existindo diversos estudos avaliando a competência de estudantes de Medicina ao realizarem exames de ultrassom (US), ainda não existem artigos avaliando seus desempenhos ao realizarem a avaliação da artéria carótida^15–19^ . Dessa maneira, este estudo teve como objetivo avaliar se estudantes de Medicina do quarto ano, após treinamento prévio, têm a capacidade de mensurar de forma correta a altura da bifurcação carotídea utilizando um aparelho de US portátil^15,20,21^ . Além disso, este artigo realizou análises estatísticas sobre as medidas obtidas entre pessoas do sexo feminino e masculino.

## MÉTODOS

Este é um estudo observacional, não intervencionista, que buscou comparar a variação da altura da bifurcação carotídea bilateralmente entre o sexo masculino e feminino. Além disso, investigou se, após treinamento prévio de 60 horas, alunos de Medicina são capazes de identificar e realizar a correta aferição da altura da bifurcação carotídea, adquirindo resultado similar a profissionais ecografistas vasculares.

Durante o período de um mês, seis alunos de Medicina do quarto ano de uma universidade do Paraná participaram de aulas sobre a ultrassonografia vascular portátil. As aulas foram ministradas por dois cirurgiões vasculares do estado, totalizando 40 horas teóricas e 20 horas práticas. Após o treinamento, foi realizada uma prova individual com 15 voluntários, na qual cada examinador realizou a técnica de medida da distância do lobo da orelha até o local em que artéria carótida comum dá origem às artérias carótida externa e interna e, ao final, as medidas foram comparadas às obtidas pelo professor orientador (Figura 1). Foi instituído que duas ou mais discrepâncias de valor maior ou igual a 0,3 cm ou uma discrepância com valor maior ou igual a 0,5 cm invalidaria a participação do pesquisador na coleta de dados. Ao fim da prova de validação, todos os seis participantes foram considerados aptos. O número de examinadores ideal indicou que apenas dois participantes já eram suficientes, mas foi decidido incluir os seis alunos, para maior aprendizado.

**Figura 1:** Imagem demonstrando como foi feita a medida da bifurcação carotídea em relação ao lóbulo da orelha.

Após essa primeira fase de capacitação, o cálculo de tamanho amostral indicou que pelo menos 280 voluntários eram necessários no estudo. Sendo assim, o grupo de estudantes realizou a mesma técnica da medida em 301 participantes: todos sentados, com a postura ereta e cabeça levemente fletida. A posição do paciente foi definida após um pré-teste, que não demonstrou diferença de medida entre o paciente sentado e deitado. Todos os participantes tinham de retirar os brincos localizados no lóbulo da orelha e, além disso, pacientes que utilizassem alargadores seriam excluídos da pesquisa (Figura 2). Foi definido que, durante todo o estudo, todas as medidas realizadas pelos alunos seriam comparadas ao resultado obtido por um profissional cirurgião vascular habilitado em ecografia vascular, o qual estaria aguardando em uma sala separada e não saberia qual o resultado encontrado pelos alunos. Caso existisse alguma discrepância maior que 0,2 cm, seria convocado mais um profissional médico, também especialista em ultrassonografia vascular, para realizar uma terceira medida. Caso a distância obtida entre os dois médicos apresentasse diferença maior que 0,2 cm, os dois resultados seriam somados e então divididos por dois para se obter a média da distância.

**Figura 2:** Imagem demonstrando o *flowchart*, com inclusão, exclusão e perda dos participantes do estudo. BAC = bifurcação da artéria carótida; US = ultrassom.

É importante salientar que os médicos eram cirurgiões vasculares e ambos possuíam certificado de habilitação em ecografia vascular com Doppler emitido pelo Colégio Brasileiro de Radiologia e Sociedade Brasileira de Angiologia e Cirurgia Vascular.

Todos os 301 participantes foram selecionados randomicamente com o auxílio de um *software* estatístico que atribuiu, de forma aleatória, um número a todos os estudantes de uma universidade do Paraná, seguindo o critério da voluntariedade e da anuência com o Termo de Consentimento Livre e Esclarecido, após aprovação da pesquisa no comitê de ética (Certificado de Apresentação de Apreciação Ética: 60822422.6.0000.0093 e Número de Aprovação: 5.997.677). A entrada de dados foi sequencial por data e ordem da realização do exame. Além disso, as duas bifurcações carotídeas de cada participante foram medidas e incluídas no estudo.

Foi decidido que, nos casos em que somente uma BAC fosse identificada ou que apresentasse visualização duvidosa, os dados seriam excluídos da análise.

Todas as artérias carótidas foram avaliadas pelo mesmo aparelho de US portátil, da marca Phillips, modelo Lumify, utilizando o *probe* linear. A técnica consistiu em identificar a artéria carótida comum e seguir seu trajeto distal até a localização de origem das artérias carótida externa e interna. O local imediatamente anterior à origem dos dois vasos foi o ponto utilizado para a medida. Caso surgissem dúvidas entre a identificação de veias e artérias, manobras de compressão seriam realizadas (Figuras 3 e 4).

**Figura 3:** Imagem ecográfica em Doppler colorido de fluxo transversal evidenciando as artérias carótidas interna esquerda, carótida externa esquerda e veia jugular esquerda.

**Figura 4:** Imagem ecográfica em corte transversal evidenciando a bifurcação carotídea esquerda. São analisadas a artéria carótida externa esquerda e carótida interna esquerda.

Foram coletadas as letras iniciais do nome e o sexo dos participantes, além da idade e das medidas bilaterais. Os oito casos nos quais as medidas obtidas entre os alunos e o ecografista diferiram em mais de 0,2 cm também foram anotados. O teste *t* de Student foi usado para as variáveis quantitativas, enquanto o teste de Fisher foi utilizado para as variáveis categóricas. A significância estatística foi considerada como p < 0,05.

## RESULTADOS

Durante todo o estudo, apenas oito medidas das 602 realizadas necessitaram de intervenção de um segundo profissional médico para realizar a nova aferição. Entre essas, todas apresentaram medida com diferença menor ou igual a 0,2 cm entre os dois profissionais médicos, não necessitando realizar a média das distâncias entre os dois ecografistas.

Entre as 602 carótidas (301 indivíduos), 332 foram de mulheres (55,1%) e 270 de homens (44,9%). A média de idade dos participantes foi de 23,2 anos, com 245 indivíduos (81,4%) entre 18 e 25 anos, e 56 (18,6%) com mais de 25 anos. A média da altura da bifurcação carotídea no lado direito foi de 5,9 cm, enquanto no lado esquerdo foi de 5,8 cm, com uma diferença média entre os lados de 0,8 cm (Tabela 1).

**Tabela 1:** Características gerais do estudo.

**Características gerais (n = 301)**		
Sexo	**n**	**%**
Feminino	166	55,1
Masculino	135	44,9
Idade classes (anos)		
18-25	245	81,4
>25	56	18,6
Idade (anos)		
Média ± DP	23,2	± 3,7
Direita		
Média ± DP	5,9	± 1,3
Esquerda		
Média ± DP	5,8	± 1,2
Diferença		
Média ± DP	0,8	± 0,1

DP = desvio-padrão.

A distância do lobo da orelha até a bifurcação carotídea foi maior em homens do que em mulheres. No lado direito, a média foi de 5,6 cm para mulheres e 6,3 cm para homens (p < 0,0001). No lado esquerdo, a média foi de 5,4 cm para mulheres e 6,2 cm para homens (p < 0,0001). A maior distância encontrada foi em um participante do sexo masculino, apresentando 9,5 cm, enquanto a menor foi de 3 cm em uma participante mulher. A variação da distância entre os lados, comparando os sexos, não mostrou significância estatística (p = 0,776) (Tabela 2). A maior diferença observada foi em uma mulher, com 8,5 cm no lado esquerdo e 4 cm no lado direito. A análise da idade dos participantes, dividida em dois grupos (18-25 anos e > 25 anos), não apresentou resultados significativos para a variação da altura da bifurcação (p = 0,052).

**Tabela 2:** Comparação entre sexo feminino e masculino.

	**Feminino**		**Masculino**		**Valor de p**
	**Média**	**± DP**	**Média**	**± DP**	**0,037**
Idade (anos)	22,4	3,6	24,3	3,8	< 0,0001
Direita	5,6	1,1	6,3	1,3	< 0,0001
Esquerda	5,4	1,1	6,2	1,3	0,776
Diferença	0,7	0,1	0,8	0,1	
Idade classes (anos)	n	%	n	%	
18-25	142	85,5	103	76,3	0,052
> 25	24	14,5	32	23,7	

DP = desvio-padrão.

## DISCUSSÃO

Embora os seis alunos de Medicina que participaram da pesquisa não tenham nenhum certificado em ecografia vascular, eles foram capazes de obter medidas da BAC muito similares a profissionais treinados e qualificados. Esse resultado foi decorrente de um intenso treinamento, tanto teórico como prático, compreendendo 60 horas no total, além de dedicação individual. Esse resultado fomenta a discussão de que a ultrassonografia não é um exame que só deve ser ensinado para médicos formados ou durante residências médicas que o incluam na grade horária. Contudo, o Ministério da Educação e Cultura brasileiro não obriga uma carga horária mínima de aulas sobre ecografia nas faculdades de Medicina, o que faz com que muitos médicos se formem sem ter a familiaridade de realizar simples exames ultrassonográficos, muitas vezes de poucos minutos, mas que podem alterar o desfecho clínico de pacientes, especialmente em ambientes de unidade de terapia intensiva (UTI) ou emergência^22-25^ . Por outro lado, as diversas universidades ao redor do mundo que já incorporaram o treinamento em ultrassonografia em seus currículos encontraram uma melhora significativa no aprendizado, motivação e acurácia do exame físico quando o treinamento com US é incluído tanto na parte clínica quanto na anatômica do curso de Medicina^16-18,26-28^ .

A constatação de que estudantes de Medicina previamente treinados são capazes de medir a altura da BAC com precisão comparável à de profissionais experientes tem limitada aplicabilidade prática. Isso ocorre porque essa medida é apenas uma parte do protocolo de ultrassonografia da artéria carótida, e os estudantes não possuem certificação para emitir laudos desse exame. No entanto, o estudo levanta a questão sobre quais outros exames poderiam ser realizados com precisão satisfatória pelos alunos. Embora a técnica de mensuração da altura da BAC seja simples, ela exige conhecimento anatômico, o que também é crucial na identificação das veias jugulares internas. Este é um passo fundamental para a realização de acessos venosos centrais guiados por US, um procedimento avançado que pode ser executado por médicos sem certificação específica.

O Brasil é um país marcado por grande heterogeneidade, com escassez de recursos materiais, profissionais qualificados e exames de imagem, especialmente fora dos grandes centros urbanos. Os médicos que optam por atuar em regiões do interior frequentemente enfrentam a falta de suporte adequado. Embora o ideal seja contar com um ultrassonografista especializado para realizar os exames, essa realidade nem sempre é viável em áreas remotas. Propõe-se, então, que os médicos recém-formados sejam capacitados a realizar exames ultrassonográficos básicos em situações de emergência, principalmente em locais onde não há outro profissional qualificado disponível. Esses exames podem ser decisivos para o desfecho clínico do paciente e, em muitos casos, representam a única opção diagnóstica disponível devido à carência de recursos^22-25^ .

Não se poderia falar de US sem comentar sobre a técnica de US à beira do leito, ou POCUS (*point-of-care ultrasound*) em inglês, que consiste na realização de um exame ultrassonográfico no local em que o paciente está, seja na UTI ou mesmo em sua residência, para diagnósticos e avaliações rápidas. Pela rapidez e facilidade em encontrar resultados que alterem a conduta de pacientes, seu uso vem sendo incorporado cada vez mais à prática médica. O exame realizado neste estudo se enquadra nessa técnica, fazendo com que o presente artigo seja mais uma base de dados a indicar que alunos de Medicina são altamente capazes de realizarem e interpretarem suas imagens^19,29–31^ . É provável que, num futuro não muito distante, a utilização do US à beira do leito seja um requisito desejável na formação médica. Diversas provas de título de especialidade, no Brasil e mundo afora, já estão incluindo como parte de sua avaliação um exame de POCUS. Ademais, muitos médicos já acreditam que com a evolução dos aparelhos ultrassonográficos, que atualmente podem ser conectados a *smartphones* ou *tablets*, o uso desse procedimento se tornará mais um pilar do exame físico, acompanhado de inspeção, palpação, percussão e ausculta^32^ .

Analisando os resultados encontrados das medidas da altura da BAC, esses foram similares aos encontrados na maioria dos estudos disponíveis na literatura internacional e latino-americana, incluindo os que utilizaram ultrassonografia, angiotomografia computadorizada, dissecção cadavérica e incisão cirúrgica^8-10,13,33,34^ . Observando apenas os resultados dos estudos que incluíram outros marcos anatômicos como referencial para avaliar a altura da BAC, os resultados encontrados também foram similares quando se identificaram à qual vértebra cervical as distâncias estudadas se referem. Dessa forma, esse é mais um estudo que corrobora a ideia de que a maioria das BACs se encontram entre C3-C4 e que pessoas do sexo feminino tendem a ter bifurcações mais altas, ou seja, mais distais em relação à origem da carótida comum. É válido ressaltar que a idade dos participantes não demonstrou influência significativa na distância da BAC, assim como em nenhum dos estudos analisados. A similaridade dos resultados, especialmente com estudos realizados em populações de diferentes origens, como América Latina, é importante considerando que a morfologia humana pode variar entre povos devido a diferenças étnicas e geográficas^14,33,35^ . Esses resultados, similares aos da literatura, reforçam ainda mais a ideia da correta técnica de medida realizada pelos alunos de Medicina.

A real importância de saber a localização da BAC é notada principalmente nos procedimentos cirúrgicos, em especial na endarterectomia, em cateterizações e em dissecções radicais no pescoço^14,35-37^ . Sua maior relevância é prever a dificuldade ou até a impossibilidade da realização da endarterectomia, cirurgia que consiste na remoção de placas de ateroma da parede carotídea e que é considerada tratamento padrão-ouro para estenose de carótida. Tal técnica não é recomendada para bifurcações acima de C2, nas quais a utilização de *stents* é preferível^14,34-38^ . Dessa forma, um dos motivos da importância de saber a altura da BAC e quais variações anatômicas podem ocorrer nesse local é que bifurcações mais altas podem exigir técnicas alternativas ou até mesmo o deslocamento da mandíbula durante a cirurgia^39^ . É válido ressaltar que, embora o sexo feminino tenha demonstrado a distância do lobo da orelha à BAC menor, nenhuma bifurcação acima de C3 foi encontrada.

Alguns questionamentos que merecem reflexão envolvem a justificativa prática e econômica de treinar estudantes de Medicina em exames de US que requerem formação especializada e cuja precisão depende amplamente da experiência do examinador, não garantindo um resultado final confiável. Questiona-se, ainda, a relevância de ensinar aos estudantes a identificação da altura da bifurcação carotídea, um conhecimento de interesse mais específico para cirurgiões vasculares. Além disso, pouco se sabe sobre a real utilidade de incluir o estudo de variações anatômicas raras no currículo médico, dada sua limitada aplicabilidade na prática clínica.

Diversas limitações estão presentes neste estudo. A primeira é a idade média dos participantes (23,2 anos), não avaliando pessoas com extremos de idades. Outro ponto digno de ser abordado é que o estudo não coletou dados como: índice de massa corporal, altura ou superfície corpórea, os quais poderiam ser considerados fatores de confusão ou modificadores de efeito. Ademais, o fato de o lóbulo da orelha depender de diversos fatores para oferecer uma medida precisa (como postura do examinador e examinado, diferenças anatômicas ou genética), pode haver uma maior chance de erros de coleta, principalmente por ter sido realizada por alunos de graduação em Medicina.

## CONCLUSÃO

Em conclusão, estudantes de Medicina, após um treinamento adequado, são capazes de realizar medições precisas da altura da BAC utilizando ultrassonografia portátil. Sobre as medidas encontradas, mulheres tendem a apresentar bifurcações carotídeas mais próximas do lobo da orelha em comparação aos homens, e a diferença entre os dois lados não apresentou diferença significativa entre os sexos.
